# How income inequality affects the subjective well-being of rural residents?

**DOI:** 10.3389/fpubh.2025.1496764

**Published:** 2025-03-26

**Authors:** Chuyue Guo, Fusheng Zeng, Dongping Xia

**Affiliations:** ^1^Economic College, Hunan Agricultural University, Changsha, China; ^2^College of Public Administration and Law, Hunan Agricultural University, Changsha, China

**Keywords:** income inequality, rural residents, subjective well-being, social mentality, ordered probit model

## Abstract

Since the initiation of economic reforms and opening-up, China’s economy has achieved remarkable growth, leading to a significant improvement in the standard of living for its people. However, the trickle-down effect of this growth has not been equitably distributed across all segments of society. This study attempts to analyze the subjective well-being (SWB) of Chinese rural residents using data from the 2021 Chinese General Social Survey (CGSS). An ordered probit (OProbit) model is constructed to investigate the impact of income inequality on the subjective well-being of Chinese rural residents. The findings reveal three key insights: (1) the benchmark regression demonstrates a significant negative impact of income inequality on the subjective well-being of Chinese rural residents. (2) Social mentality emerges as a critical mediating channel through which income inequality undermines subjective well-being. (3) The impact of income inequality on subjective well-being varied significantly depending on factors such as age, gender, and marital status. As enhancing well-being gains increasing recognition as a central goal in global public health policy, the findings of this study provide valuable insights for designing policies aimed at improving subjective well-being, particularly in rural contexts.

## 1 Introduction

Happiness is the ultimate goal pursued by all mankind and the aim of economic development in countries around the world. As the world’s largest developing country, China has achieved a miracle of rapid economic growth. China’s GDP surpassed the 100 trillion yuan threshold for the first time in 2020, cementing its position as the world’s second-largest economy by size. However, while achieving tremendous success in the economic sphere, studies indicate that the subjective well-being (SWB) of Chinese citizens has not seen a corresponding increase. According to the latest World Happiness Report (1), although material standards, quality of life, and life expectancy have significantly improved and surpassed the global average, the happiness index of Chinese residents has not risen markedly. This mismatch between the growth of residents’ subjective well-being and socioeconomic development across various domains is termed the “Easterlin Paradox.” With accumulating research, the existence of this paradox in China has been consistently validated (2, 3).

Why has not the progress of socioeconomic development brought about higher levels of happiness among residents? Many studies have found that income inequality plays an important role in it, and pointed out that the deep level of income inequality is a significant reason for the existence of the “Easterlin paradox” in China (4, 5). According to data released by the National Bureau of Statistics of China (6), the Gini coefficient of income distribution in China has remained in the range of 0.46–0.47 in recent years, which is not only significantly higher than the benchmark level of 0.24–0.36 in developed countries, but also consistently high among the ranks of moderately developed economies. Data comparison shows that the trickle-down effect of current economic development has failed to permeate all levels of society. This structural imbalance distributing development outcomes has led to a persistent income gap problem. From the perspective of the Chinese context, on the one hand, the wealth created by economic growth has not been evenly distributed among its citizens, and the income distribution gap among residents has remained high, which has become an important factor restricting people from obtaining more happiness. On the other hand, as a social phenomenon that residents can directly perceive, the continuous expansion of income inequality and wealth gap will undoubtedly have an impact on the subjective indicator of residents’ happiness (7).

As a relatively vulnerable group in society, the subjective well-being level of rural residents is a key focus of social attention. From the perspective of overall income inequality in rural areas of China, the per capita disposable income of high-income households (up to 20%) was about 3.3 times that of low-income households (down to 20%) in 2010, which expanded to 9.46 times in 2016. Although it has decreased in recent years, it is still at a high level. Therefore, this article delves into the impact mechanism of income inequality on the happiness of China’s rural residents, which helps to improve the theoretical explanation of the “happiness paradox” in development economics and has important academic value and practical significance (8).

## 2 Literature review

After reviewing the literature on income inequality and subjective well-being, scholarly discourse reveals three predominant theoretical positions regarding their correlation. The first view is that there is a negative correlation between these two factors. Based on the theory of “relative deprivation,” some scholars believe that income inequality leads to relative deprivation of residents, thereby reducing subjective well-being (9–11). With the deepening of the research, scholars also found that income inequality is a crucial reason leading to the decline of residents’ income satisfaction (12–14). In economies with low income inequality, the marginal positive effect of a 10% increase in income on life satisfaction is more than twice as strong as in high inequality economies (15). Moreover, With the improvement of income distribution fairness, the marginal improvement effect of unit income growth on residents’ happiness shows an increasing trend (16, 17). In addition, other scholars support that income inequality is negatively related to residents’ happiness, but the size of this effect varies by region and different groups (16, 18). As Tim suggests, in countries with higher levels of overall income inequality, those who are able to make upward and downward comparisons are happier. However, in countries with low levels of inequality, this effect does not exist (19). Yong ma also pointed out that in different regions of China, income inequality has different effects on residents’ happiness (20).

The second view is that income inequality can increase residents’ subjective well-being. It was Hirschman and Rothschild who first used the “tunnel effect” theory to explain the positive impact of income inequality on residents’ subjective well-being (21). The “tunnel effect” refers to the feeling of frustration when driving in a two-lane tunnel where two lanes are blocked. But if cars on one lane start driving, drivers on the other lane will feel happy, which means they can also move quickly. Similarly, income inequality can be seen as a signal of economic opportunity, and the existence of income inequality can increase the individual perceptions of upward social mobility, thereby increasing happiness (22).

The third view is that income inequality has no effect on residents’ subjective well-being. Although few scholars support this view, Lane Kenworthy proposes that income inequality has no significant impact (negative or positive) on residents’ subjective happiness after studying both developing and developed countries (23). Moreover, Berg and Veenhoven found little relationship between income inequality and national average happiness (24).

Compared with the existing literature, this study has achieved three breakthroughs in theoretical innovation and methodology, which are reflected in the following dimensions. Firstly, in terms of research perspective, existing literature mostly focuses on the happiness of urban residents or older adult groups (25, 26), while this article breaks through by positioning the research object on vulnerable rural groups, echoing the core concerns of inclusive development theory. Secondly, at the level of econometric methods, this study innovatively uses geographic information data to construct instrumental variables, using the nearest straight-line distance from the local area to the port as the instrumental variable. Through the conditional mixed process (CMP), it effectively overcomes the problems of bidirectional causality and omitted variables in the model. Again, in the dimension of mechanism analysis, this article constructs a mediating transmission model of “income inequality—social mentality—happiness,” which improves the research on the mechanism of income inequality on rural residents’ happiness. Above innovations have achieved a systematic breakthrough in the research of happiness economics in three dimensions: theoretical construction, methodological innovation, and mechanism analysis.

## 3 Materials and methods

### 3.1 Study sample

This study utilizes data from the Chinese General Social Survey (CGSS), a nationally representative survey conducted by Renmin University of China since 2003. The CGSS, recognized as China’s first comprehensive and continuous academic survey project, collects data from over 10,000 households across all provinces, municipalities, and autonomous regions in mainland China. The analytical framework utilizes the latest publicly available iteration (CGSS2021), which encompasses detailed information on population demographics, family structure, health status, and income levels for the year 2020.

To align with the research objectives, the data processing involved several key steps. Firstly, urban resident samples were excluded to focus exclusively on rural populations. Secondly, samples with missing critical information or outliers were removed. Finally, a mixed cross-sectional dataset comprising 2,776 rural residents from 19 Chinese provinces was retained for empirical analysis^1^. This refined dataset ensures the study’s focus on rural demographics while maintaining data quality and relevance.

### 3.2 Measures

The explained variable in this paper is subjective well-being of China’s rural residents. This variable is derived from the CGSS2021 questionnaire item: “Do you feel that you are in high subjective well-being in your life?” Respondents were provided with five response options: very low subjective well-being, relatively low subjective well-being, general, relatively high subjective well-being, and very high subjective well-being, which were assigned numerical values of 1 to 5, respectively. The use of this single-item measure is widely adopted in related studies due to its validity, reliability, and consistency across repeated trials, making it a robust indicator for assessing subjective well-being (27).

The key explanatory variable is income inequality. Drawing on the methodology proposed by D’Ambrosio et al. (28), this study measures income inequality among rural residents from the perspective of individual income inequality. To construct the individual income inequality index, the average annual income of each individual is first estimated using the survey question: “What was your personal total income for the whole of last year (2020)?.” Subsequently, the Kakwani Index is calculated based on the theory of individual income inequality (Equation 1), which is a measure used to assess progressivity or regressivity in taxation or social welfare systems (29). It quantifies how much a tax or transfer system redistributes income by comparing the distribution of taxes or benefits to the distribution of income. A positive Kakwani Index indicates that the system is progressive, meaning higher-income individuals pay a larger share of taxes or receive fewer benefits relative to their income. A negative Kakwani Index suggests the system is regressive, with lower-income individuals bearing a disproportionate burden or receiving fewer benefits. In line with existing literature, both the Yitzhaki Index (Equation 2) and the Kakwani Index are employed as measures of individual income inequality, with the Kakwani Index serving as the primary example in this study. Rural residents are designated as the reference group for these calculations. The individual income inequality index is constructed by comparing each individual’s income to the incomes of others in the reference group who earn more. Specifically, X stands for the reference group with sample size n and income Xn. Accordingly, Xn is sorted out in ascending order, i.e., X_1_ < X_2_ < ... <Xn, while the individual income deprivation index is used to construct the Kakwani Index.


(1) $$\mathrm{Kakwani}=\frac{1}{n\mu }\overset{n}{\underset{i=k+1}{\sum }}\left(x_{i}-x_{k}\right)$$

Here, μ denotes the mean income of the rural population, and $x_{k}$ presents the kth individual income. The Kakwani Index is calculated using the aforementioned equation, with its value ranging between [0, 1]. A higher value indicates a greater level of relative deprivation. Additionally, the Yitzhaki Index is employed as a proxy for income inequality among rural residents in the robustness analysis (30), as defined by the following mathematical expression:


(2) $$\mathrm{Yitzhaki}=\frac{1}{n}\overset{n}{\underset{i=k+1}{\sum }}\left(x_{i}-x_{k}\right)$$

Additionally, this paper employs the straight-line distance (kilometers) from the nearest port as an instrumental variable, as it is correlated with the endogenous variable (income inequality) but does not exert a direct influence on happiness, thus meeting the necessary criteria for a valid instrument. Besides, social mentality is used as a mediator variable, which presents the attitude that residents articulate toward social events and concerns based on the living conditions and social system. Aligned with the existing literature (31), this research paper argues that social mentality includes residents’ perception of present social fairness and assessment of social trust. This study weights social trust (X1) and social fairness (X2) to produce a social mentality index (Equation 3). Notably, the social mentality index strives to capture the social mentality of rural residents in a more comprehensive and accurate manner. The weight of the two is equal since it is difficult to distinguish which of the two variables exerts a stronger influence on the social mentality index of rural residents. Correspondingly, the social mentality index is as follows:


(3) $$\mathrm{Socialmentality}=\left(x1+x2-2\right)/8$$

The control variables primarily encompass individual and family characteristic variables. Individual characteristics include party membership status, ethnicity, age (along with its squared term), marital status, gender, religious affiliation, and residential status. Family characteristics, consist of the number of family-owned houses, household size, and the number of private cars owned by the family. These variables collectively provide a comprehensive framework for analyzing the factors influencing the outcomes under study (Table 1).

**Table 1:** Descriptive statistics of the main variables.

Types	Var. name	Value and meaning	Std. dev	Min	Max
Explained variables	Subjective Well-Being of Rural Residents	Very bad = 1, bad = 2, neutral = 3, good = 4, very good = 5	0.85	1.00	5.00
Key explanatory variable	Kakwani Index	Income inequality	0.20	0.00	1.00
	Yitzhaki Index	Income inequality	7437.08	0.00	36989.67
Instrumental variables	Distance	The straight-line distance from the nearest port (kilometers)	341.91	6.58	1268.73
Mediator variable	Social Fairness	Completely unfair = 1, Relatively unfair = 2, not fair but not unfair = 3 Relatively fair = 4, Completely fair = 5	1.00	1.00	5.00
	Social Trust	strongly disagree = 1, Disagree = 2, Not agree = 3, Quite agree = 4, Strongly agree = 5	1.01	1.00	5.00
	Social Mentality	(Social Fairness + Social Trust-2)/8	0.21	0.00	1.00
Control variables	Age	Age in 2020	16.56	18.00	91.00
	Age^2^	age × age	16.94	3.24	82.81
	Gender	Male = 1, Female = 0	0.50	0.00	1.00
	Party membership status	Party members = 1, otherwise = 0	0.25	0.00	1.00
	Marital status	With spouse = 1, otherwise = 0	0.42	0.00	1.00
	Religious affiliation	With religious affiliation = 1, otherwise = 0	0.28	0.00	1.00
	Ethnicity	Han nationality =1, otherwise = 0	0.28	0.00	1.00
	Residential status	Rural = 1, Township = 2, County town = 3, Suburban = 4, city = 5	1.93	1.00	5.00
	Family-owned houses	Number of houses owned	0.64	0.00	6.00
	Family-owned Cars	Family owning a car/cars = 1, no car = 0	0.49	0.00	1.00
	Household size	Number of family members	1.26	0.00	13.00

### 3.3 Model settings

#### 3.3.1 OProbit model

In this study, it employs the ordered probit (OProbit) model to examine the impact of income inequality on rural residents’ subjective well-being, as the dependent variable is an ordered categorical variable of 1–5 with inherent discrete and hierarchical properties. The OProbit framework is theoretically appropriate for such ordinal outcomes, as it explicitly models latent variable thresholds to capture nonlinear transitions between adjacent well-being categories while constraining predicted probabilities within the valid [0, 1] range. In contrast, commonly used ordinary least squares (OLS) regression, though computationally simple, violates critical statistical assumptions by treating the outcome as continuous and normally distributed. This mismatch leads to biased coefficient estimates, invalid significance tests, and nonsensical predictions. The basic principle of OProbit model is as follows (Equation 4):


$$P\left(y=0/x\right)=P\left(y^{*}\leq r_{0}|x\right)=\emptyset \left(r_{0}-x'\beta \right)$$

$$P\left(y=1/x\right)=P\left(r_{0}\leq y^{*}\leq r_{1}|x\right)=\emptyset \left(r_{1}-x'\beta \right)$$

(4) $$P\left(y=J/x\right)=1-\emptyset \left(r_{J-1}-x'\beta \right)$$

Therefore, combined with the research objects of this paper, the basic form of the model can be set as follows (Equation 5):


(5) $${SWB}_{i}=\alpha +\beta {\mathrm{Inequality}}_{i}+\gamma {CV}_{i}+\mu_{i}$$

Where *i* stands for the individuals, and ${SWB}_{i}$ presents the explained variable in this study, which represents subjective well-being of rural residents in China. Moreover, ${\mathrm{Inequality}}_{i}$ serves as the core explanatory variable of income inequality. It reflects the income inequality of Chinese rural residents. Meanwhile, ${CV}_{i}$ represents the control variables, including individual and family characteristic variables. $\mu_{i}$ captures the random disturbance term.

#### 3.3.2 CMP model

To mitigate the issue of reverse causality between the income inequality and SWB, the instrumental variable approach is used to deal with endogeneity to some extent. Due to the fact that the explained variable in this article is an ordered categorical variable, the traditional two-stage least squares method (IV-2SLS) cannot accurately handle its nonlinear features. Therefore, a more suitable method is needed for endogeneity testing and parameter estimation. This study employs the conditional mixed process (CMP) method to address potential endogeneity issues in the model. This method, proposed by Roodman (32), is based on the maximum likelihood estimation approach and achieves the estimation of a two-stage regression model by constructing a recursive system of equations. The core of this method lies in the selection of appropriate instrumental variables, and the estimation process involves a two-stage regression analysis: In the first stage, instrumental variables for the core explanatory variables are identified, and their correlations are estimated. In the second stage, these instrumental variables are incorporated into the baseline model for regression, and the exogeneity of the core explanatory variables is assessed by referring to the endogeneity test parameter (atanhrho_12). If the parameter value significantly differs from 0, it indicates the presence of endogeneity issues in the model, suggesting that the CMP method’s estimation results are superior. Conversely, if the parameter value does not significantly differ from 0, it implies that the baseline model’s estimation results are reliable.

#### 3.3.3 KHB model

To further explore the mechanism through which income inequality influences the subjective well-being of rural residents, this study adopts the KHB mediation effect method to analyze the mediating effects. In this research, the dependent variable subjective well-being is an ordered categorical variable. The KHB model employs a standardized coefficient decomposition method, which eliminates the bias caused by scale variations in the dependent variable within nonlinear models (e.g., Ordered Probit). This ensures unbiased advantages in calculating the proportion of mediation effects (33).

The analysis is grounded in two key variables: social trust and social fairness, which are standardized and aggregated to construct the social mentality index as the mediating variable. Social trust and perceptions of social fairness serve as critical indicators of an individual’s social mentality. By integrating these dimensions, the study formulates a composite mediator variable, referred to as the social mentality index, to comprehensively capture the underlying social psychological dynamics. We used the following (Equation 6):


$${SWB}_{i}=\alpha_{11}+\beta_{11}\;{\mathrm{Inequality}}_{i}+\gamma_{11}{CV}_{i}+\mu_{1i}$$

$${SWB}_{i}=\alpha_{12}+\beta_{12}\;{\mathrm{Inequality}}_{i}+\gamma_{12}{CV}_{i}+\delta {\mathrm{Mediator}}_{i}+\mu_{2i}$$

(6) $$\mathrm{Residual}={\mathrm{Mediator}}_{i}-\left(\alpha_{12}+\beta_{12}{\mathrm{Inequality}}_{i}+\gamma_{12}{CV}_{i}\right)$$

Where ${\mathrm{Mediator}}_{i}$ means the social mentality index, which is calculated from social trust and social fairness according to Equation 3, and ${CV}_{i}$ reflects the control variable.

## 4 Results

### 4.1 Benchmark results

Table 2 presents the results of the benchmark ordered probit (OProbit) regression. After controlling for the basic characteristics of rural residents and household attributes, income inequality is found to exert a statistically significant negative impact on the subjective well-being of rural residents. This indicates that income inequality undermines the subjective well-being of rural residents, thereby diminishing their overall happiness. As illustrated in Model (1) of Table 2, income inequality exhibits a statistically significant negative impact on the subjective well-being of rural residents, with a correlation coefficient of −0.4016, in the absence of control variables. In Model (2), after incorporating individual characteristics as control variables, the negative correlation between income inequality and rural residents’ subjective well-being remains significant, with the correlation coefficient increasing to −0.3736. Furthermore, in Model (3), which introduces additional control variables for household characteristics on the basis of Model (2), the correlation coefficient between income inequality and rural residents’ subjective well-being is estimated at −0.3359, maintaining its statistical significance. It can be seen that the subjective well-being of rural residents significantly decreases by 0.079 standard deviation for every 1 unit standard deviation increase in income inequality. These results consistently demonstrate that income inequality adversely affects the subjective well-being of rural residents across different model specifications. Among the control variables, marital status and age within the individual characteristics, as well as private vehicle ownership within the household characteristics, significantly influence the subjective well-being of rural residents. Specifically, age exhibits a statistically significant negative association with the well-being of rural residents, indicating that older residents tend to report lower levels of subjective well-being. Concurrently, the coefficient for the quadratic term of age (age^2^) is positive, suggesting a non-linear relationship between age and well-being over the life cycle, consistent with a U-shaped pattern (34). Furthermore, rural residents with spouse report significantly higher levels of well-being compared to those without spouse. Similarly, household ownership of a private vehicle is positively associated with the happiness of rural residents, highlighting its role in enhancing subjective well-being.

**Table 2:** OProbit regression results.

	(1)	(2)	(3)
Variable	SBW		
Kakwani Index	−0.4016***	−0.3736***	−0.3359***
	(−3.501)	(−3.133)	(−2.793)
Age		−0.0740***	−0.0739***
		(−8.368)	(−8.337)
Age^2^		0.0008***	0.0008***
		(8.760)	(8.880)
Gender		−0.0032	0.0061
		(−0.072)	(0.137)
Ethnicity		0.0737	0.0928
		(1.046)	(1.304)
Party membership status		0.1686**	0.1506**
		(2.472)	(2.183)
Marital status		0.2814***	0.2466***
		(5.060)	(4.347)
Religious affiliation		0.0315	0.0268
		(0.378)	(0.317)
Residential status		0.0250	0.0161
		(0.682)	(0.435)
Household size			0.0272**
			(2.370)
Family-owned houses			0.0019
			(0.043)
Family-owned Cars			0.1936***
			(4.306)
Observations	2,776	2,776	2,776
Pseudo R2	0.105	0.116	0.125

Robust *t*-statistics in Parentheses: ***p < 0.01, **p < 0.05, *p < 0.1.

### 4.2 Robustness checks

Replacement of Regression Methods: since the explained variable subjective well-being of rural residents is an ordered categorical variable, this study also introduces ordinary least squares (OLS) and ordered logit (OLogit) to regress the two. Accordingly, the regression results are exhibited in model (1) and model (2) of Table 3. The results of both the OLS and OLogit models illustrate that income inequality still exerts a significant negative influence on the subjective well-being of rural residents. It shows that the above results are reliable and robust.

**Table 3:** Robustness test results.

	(1)	(2)	(3)	(4)	(5)
	OLS	OLogit	OLS	OProbit	OLogit
Variable	SWB				
Kakwani Index	−0.2906***	−0.5573***			
	(−3.230)	(−2.643)			
Yitzhaki Index			−0.0000**	−0.0000**	−0.0000**
			(−2.404)	(−2.090)	(−2.008)
Control variables	YES	YES	YES	YES	YES
Observations	2,776	2,776	2,776	2,776	2,776
Pseudo R2	/	0.1218	/	0.0221	0.0218
R2	0.046	/	0.0497	/	/

Robust *t*-statistics in Parentheses: ***p < 0.01, **p < 0.05, *p < 0.1.

Replacement of Explanatory Variables: As demonstrated in Table 3, Models (3)–(5) employ the Yitzhaki Index as an alternative measure to the Kakwani Index in the empirical analysis. Notably, the regression outcomes consistently reveal a statistically significant negative association between income inequality (proxied by the Yitzhaki Index) and subjective well-being among rural residents. This persistence of significance across alternative model specifications confirms the robustness of our core findings against measurement variations. Furthermore, to ensure methodological rigor, this paper implemented a tripartite estimation strategy utilizing OLS, OProbit, and OLogit models. The congruence of statistically significant coefficients across all three estimation techniques substantiates the reliability of our conclusion regarding the welfare-depressing effect of income inequality. These robustness checks through both alternative inequality metrics and diverse econometric approaches collectively reinforce the empirical validity of our central thesis.

### 4.3 Endogenous analysis

The estimated coefficients may be biased due to endogeneity issues arising from omitted variables, reverse causality, and measurement errors. For instance, unobserved confounders in the model may influence both income inequality and rural residents’ subjective well-being. Simultaneously, bidirectional causality could exist: while income inequality affects well-being, perceived happiness might reciprocally shape individuals’ economic behaviors and regional income distribution patterns. Additionally, systematic measurement errors are plausible, as self-reported income data may suffer from underreporting or recall biases, while subjective well-being metrics derived from survey instruments are inherently susceptible to response heterogeneity. To address these identification challenges, this study implements an instrumental variable (IV) approach. Therefore, this article uses the instrumental variable method to address endogeneity issues. The nearest distance from each province to a coastal port is chosen as the instrumental variable. The instrumental variable (distance to port) satisfies the condition that it is related to the endogenous explanatory variable (income inequality) and independent of the error term. Table 4 model (1) shows the regression results of the first stage of CMP. The outcomes of the 1st-stage regression illuminate that the size of the nearest distance to the coastal port from each province has a significant positive impact on income inequality. This infers that the further distance from the coastal port, the more severe the income inequality is. Thus, revealing that the instrumental variables incorporated in this study satisfy the assumption of correlation. Additionally, the F statistic is 31.11, greater than 10, indicating that there is no weak instrumental variable issue. At the same time, the endogeneity test parameter atanhrho_12 is significantly non-zero at the 1% level, indicating the endogeneity of income inequality variables in model estimation. Table model (2) shows that the estimated coefficient of inequality is reported to be −2.1320, indicating that after overcoming endogeneity issues, income inequality still has a negative impact on the happiness of rural residents (Table 4).

**Table 4:** Endogeneity test results.

	(1)	(2)
Variable	Kakwani index	SWB
Distance	0.0001***	
	(5.579)	
Kakwani Index		−2.1320**
		(−2.531)
atanhrho_12	0.3831**	
	(2.031)	
F statistic	31.11	

### 4.4 Heterogeneities analysis

This paper runs OProbit regressions with differences in gender, age, and marriage, in order to determine whether there exists heterogeneity in terms of the influence of income inequality on the subjective well-being of rural residents. In the context of age, the WTO defines older persons as those over 60 years of age. On the one hand, income inequality does not exhibit a significant effect on young and middle-aged rural individuals (age < 60). On the other hand, it has a significant negative impact on the rural older adults (age ≥60). In terms of gender, income inequality significantly lowers the subjective well-being of rural males, but demonstrates no linkage with the well-being of the female. From the standpoint of marital status, income inequality displays no significant impact on rural residents without a spouse, whereas income inequality shows a significant negative influence on the married residents, with a correlation coefficient of −0.4303 (Table 5).

**Table 5:** Heterogeneity analysis.

	(1)	(2)	(3)	(4)	(5)	(6)
	Age < 60	Age ≥60	Female	Male	Without a spouse	With spouse
Variable	SWB					
Kakwani Index	−0.3129	−0.6066**	−0.2001	−0.5067***	−0.0173	−0.4303***
	(−0.206)	(−2.022)	(−1.247)	(−2.701)	(−0.073)	(−2.953)
Control variables	YES	YES	YES	YES	YES	YES
Observations	1792	984	1,517	1,259	693	2083
Pseudo R2	0.023	0.036	0.017	0.071	0.031	0.021

### 4.5 Analysis of mediation effect

This article further uses the KHB method to test the mediating effect, decomposing the total effect of income inequality on the subjective well-being of rural residents into direct and mediating effects, and calculating the size and contribution of the mediating path to the outcome variables. This study chooses social mentality as the mediating variable, and the composite of social equity perception and social trust as the proxy indicator. The regression outcomes are presented in Table 6. In particular, the social mentality index significantly enhances the well-being of rural residents, with a mediating path share of 24.26%.

**Table 6:** Mediation effect result.

	(1)	(2)	(3)
Variable	Social mentality	*t*	[95% conf. interval]
Total effect	−0.3594***	−2.857	[−0.606,-0.113]
Direct effect	−0.2722**	−2.163	[−0.519,-0.026]
Indirect effect	−0.0872**	−2.076	[−0.169,-0.05]
Confounding ratio	1.32		
Full model explanation	24.26%		

## 5 Discussion

### 5.1 Key findings

This study takes rural residents in China as the research subjects and explores how income inequality affects the subjective well-being of them. The study finds that income inequality has a negative impact on the subjective well-being of rural residents with a correlation coefficient of −0.3359, and is significant at the 1% statistical level. Furthermore, the research also found that the relationship between income inequality and the subjective well-being of rural residents varies by gender, marital status, and age. Firstly, in terms of gender differences, income inequality has a lower impact on rural women’s subjective well-being than rural men’s, and this is the same conclusion reached by scholar M. Niaz Asadullah (2). This is due to differences in expectations and perceptions of the income inequality between men and women. Rural women are less sensitive to the income gap and more concerned about their absolute income level. Rural men are more sensitive to income inequality and pay more attention to their relative income status rather than absolute income level. This can be explained by the theory of relative deprivation, where an individual’s sense of happiness decreases with an increase in others’ income. Secondly, from the perspective of the marital status, income inequality deprives rural married residents of their subjective well-being, while income inequality has no significant effect on rural non-married residents, because rural residents with spouses have higher family responsibilities and less optimistic expectations about the future than rural residents without spouses. Clearly, income inequality severely deprives married rural residents of their happiness. Finally, there are age differences, with income inequality not having a significant impact on the well-being of the young and middle-aged rural population, but having a negative impact on the well-being of the rural older adults. This is because income inequality can stimulate the innovative and enterprising spirit of rural young people, further promote their social mobility, and supplement their income expectations and future prospects. In contrast, older people in rural areas have a more pessimistic view of income inequality, and they tend to see it as a deterrent rather than an economic opportunity (35).

### 5.2 Add findings

This study used two methods to examine whether income inequality has a negative impact on subjective well-being of rural residents: replacement independent variables and replacement the econometric model. Firstly, this paper replaces the Kakwani index with the Yitzhaki index. The Yitzhaki index focuses more on the welfare distribution of the middle and low-income groups through the weight of income ranking. Secondly, due to independent variables being continuous variables and the dependent variable being an ordered categorical variable. Therefore, this article uses OLS and OLogit for robustness testing. Both methods show that income inequality has a significant negative impact on the happiness of rural residents in China. In addition, the CMP method is used to ascertain the question of whether income inequality and the subjective well-being of rural residents are endogenous. Finally, the KHB method is used to reveal that income inequality further affects the subjective well-being of rural dwellers by impacting their social mentality. The research results show that the mediating effect of social mentality accounts for 24.26%, indicating that income inequality can further affect the happiness of rural residents by influencing their social mentality.

### 5.3 Strengths and limitations

This research paper exhibits two notable limitations. Firstly, the dataset employed in this study is derived from the CGSS2021 survey, which primarily reflects statistical data from 2020. This results in a temporal discrepancy of approximately 4 years relative to the study’s execution, potentially compromising the timeliness and relevance of the findings. Secondly, the selection of instrumental variables in this research lacks comprehensiveness. Specifically, the social mindset index is constructed solely based on two indicators: social fairness perception and social trust. Due to data availability constraints, the index fails to incorporate a more extensive range of social-level indicators that could provide a more robust and nuanced representation. Consequently, future research should endeavor to integrate additional relevant indicators to enhance the construct validity and predictive power of the social mindset index.

## 6 Conclusion

Currently, China is in the critical implementation stage of its rural revitalization strategy, wherein addressing the “three rural issues” (agriculture, rural areas, and farmers) remains paramount. Understanding the impact of income inequality on rural residents’ subjective well-being is essential for formulating more effective policy interventions and social programs. Utilizing data from CGSS 2021, this study investigates the intrinsic relationship between income inequality and rural residents’ subjective well-being, employing a meticulously constructed income inequality index. The empirical findings demonstrate that for each standard deviation increase in income inequality, rural residents’ subjective well-being decreases by an average of 0.079 standard deviations. Specifically, social mentality emerges as a critical mediating mechanism through which income inequality adversely affects rural residents’ subjective well-being. Further heterogeneity analysis reveals nuanced patterns in the relationship between income inequality and subjective well-being across demographic groups. Income inequality exhibits no statistically significant effect on the subjective well-being of rural middle-aged and younger individuals. In contrast, it exerts a significant negative impact on the well-being of older adult rural residents. Gender-specific analysis indicates that income inequality significantly reduces the well-being of rural males, while showing no discernible effect on females. From a marital status perspective, income inequality does not significantly influence the subjective well-being of unmarried rural residents but demonstrates a pronounced negative effect on their married counterparts. Moreover, mediation analysis confirms that income inequality further influences rural residents’ subjective well-being through its impact on social mentality.

In line with the aforesaid findings of this study, the authors put forward the following recommendations:

Foremost, institutional safeguards for rural revitalization strategies must be strengthened to promote an inclusive development model that ensures the equitable distribution of economic growth dividends across all rural populations, rather than concentrating benefits among privileged elites. Specifically, through coordinated interventions by governmental agencies and social organizations, a comprehensive resource allocation mechanism should be established, prioritizing targeted policy implementations for vulnerable rural groups. Concurrently, it is imperative to systematically deconstruct the asymmetric resource distribution patterns inherent in traditional social networks (termed the “differential pattern”), by constructing formal social support systems and cultivating innovative mutual-aid paradigms to mitigate their latent adverse effects on social equity.

Secondly, a multidimensional infrastructure development strategy should be implemented through public-private partnerships, prioritizing the modernization of transportation networks, water supply systems, energy grids, and sanitation facilities. This infrastructure enhancement serves dual purposes: establishing agricultural production stabilization mechanisms and constructing income security buffers for marginalized rural populations. Concurrently, a tripartite governance framework engaging governmental entities, market stakeholders, and civil society organizations must be institutionalized to advance structural reforms in rural social service provision systems. These synergistic interventions collectively operationalize the pro-poor growth paradigm within inclusive development frameworks, where economic expansion deliberately prioritizes welfare multipliers for disadvantaged agrarian cohorts through institutionalized redistribution channels.

Finally, rationally view the deprivation of happiness of rural residents caused by income inequality. In the process of promoting rural construction, we should not only give play to the positive guiding role of “new farmers” in rural governance, but also rationally view the practical problem of income inequality of rural residents, and control income inequality within a reasonable range. By accelerating the reform of the income distribution system, we will promote the improvement of the income level and upward income mobility of rural low-income groups, so that the fruits and dividends of economic development will benefit all the people.

## Data Availability

Publicly available datasets were analyzed in this study. This data can be found at: http://cgss.ruc.edu.cn/.

## Footnotes

^1^19 provinces including Beijing, Hebei, Liaoning, Jiangsu, Zhejiang, Fujian, Shandong, Guangxi, Jiangxi, Henan, Hubei, Hunan, Shanxi, Gansu, Inner Mongolia, Chongqing, Anhui, Shaanxi, and Ningxia.

## References

[1] Helliwell J Layard R Sachs J De Neve J-E Aknin L Wang S. Happiness and age: Summary. World Happiness Report. Oxford: University of Oxford (2024). doi: 10.18724/WHR-KK3M-B586

[2] Asadullah MN Xiao S Yeoh E. Subjective well-being in China, 2005–2010: the role of relative income, gender, and location. China Econ Rev. (2018) 48:83–101. doi: 10.1016/j.chieco.2015.12.010

[3] Li L Shi L. Economic growth and subjective well-being: analyzing the formative mechanism of Easterlin paradox. J Chin Sociol.. (2019) 6:1–19. doi: 10.1186/s40711-018-0090-9

[4] Chen C Li C. Research on income inequality, redistribution preference and residents’ subjective well-being. Public Finance Res. (2016) 12:64–77. doi: 10.19477/j.cnki.11-1077/f.2016.12.006

[5] He L Pan C. Uncover the “Easterlin paradox” of China: income gap, inequality of opportunity and happiness. J Manag World. (2011) 8:11–22+187. doi: 10.19744/j.cnki.11-1235/f.2011.08.003

[6] National Bureau of Statistics of China. Yearbook. (2024). Available online at: https://www.stats.gov.cn/english/Statisticaldata/yearbook/ [Accessed February 17, 2025]

[7] Ding J Salinas-Jiménez J Salinas-Jiménez MDM. The impact of income inequality on subjective well-being: the case of China. J Happiness Stud. (2021) 22:845–66. doi: 10.1007/s10902-020-00254-4

[8] Easterlin R Mcvey L Switek M Sawangfa O Zweig J. The happiness-income paradox revisited. Proc Natl Acad Sci USA. (2010) 107:22463–8. doi: 10.1073/pnas.1015962107, PMID: 21149705 PMC3012515

[9] Wang J. Will wealth inequality decrease happiness?-empirical evidence from China. Front Psychol. (2023) 14:1259456–6. doi: 10.3389/fpsyg.2023.1259456, PMID: 38362522 PMC10867187

[10] Diener E Tay L Oishi S. Rising income and the subjective well-being of nations. J Pers Soc Psychol. (2013) 104:267–76. doi: 10.1037/a0030487, PMID: 23106249

[11] Lilly KJ Sibley CG Osborne D. Examining the indirect effect of income on well-being via individual-based relative deprivation: longitudinal mediation with a random intercept cross-lagged panel model. Intern J Psychol: J Inter de psychologie. (2024) 59:368–77. doi: 10.1002/ijop.13097, PMID: 38018263

[12] Lakshmanasamy T Maya K. The effect of income inequality on happiness inequality in India: a Recentered influence function regression estimation and life satisfaction inequality decomposition. Indian J Human Develop. (2020) 14:161–81. doi: 10.1177/0973703020948468

[13] Oishi S Kesebir S Diener E. Income inequality and happiness. Psychol Sci. (2011) 22:1095–100. doi: 10.1177/0956797611417262, PMID: 21841151

[14] Du H Huang Y Ma L Chen X Chi P King RB. Subjective economic inequality is associated with lower well-being through more upward comparison and lower trust. Appl Psychol Health Well Being. (2024) 16:25–41. doi: 10.1111/aphw.12467, PMID: 37436073

[15] Quispe-Torreblanca EG Brown GDA Boyce CJ Wood AM De Neve J-E. Inequality and social rank: income increases buy more life satisfaction in more equal countries. Personal Soc Psychol Bull. (2021) 47:519–39. doi: 10.1177/0146167220923853, PMID: 32468919 PMC7961663

[16] Hajdu T Hajdu G. Reduction of income inequality and subjective well-being in Europe. Dent Econ. (2014) 8:1–29. doi: 10.5018/economics-ejournal.ja.2014-35

[17] Lei X Shen Y Smith JP Zhou G. Life satisfaction in China and consumption and income inequalities. Rev Econ Househ. (2018) 16:75–95. doi: 10.1007/s11150-017-9386-9, PMID: 29545732 PMC5846478

[18] Oishi S Kesebir S. Income inequality explains why economic growth does not always translate to an increase in happiness. Psychol Sci. (2015) 26:1630–8. doi: 10.1177/0956797615596713, PMID: 26338882

[19] Liao TF. Income inequality, social comparison, and happiness in the United States. Socius: Sociolog Res Dynamic World. (2021) 7:2378023120985648. doi: 10.1177/2378023120985648, PMID: 40066071

[20] Ma Y Chen D. Openness, income inequality, and happiness: evidence from China. J Econ Inequal. (2022) 20:371–93. doi: 10.1007/s10888-021-09507-5

[21] Hirschman AO. The changing tolerance for income inequality in the course of economic development. World Dev. (1973) 1:29–36. doi: 10.1016/0305-750X(73)90109-5

[22] Katic I Ingram P. Income inequality and subjective well-being: toward an understanding of the relationship and its mechanisms. Bus Soc. (2018) 57:1010–44. doi: 10.1177/0007650317701226

[23] Kenworthy L. Income inequality probably has had little or no effect on subjective well-being. Soc Sci Res. (2017) 62:36–8. doi: 10.1016/j.ssresearch.2016.12.008, PMID: 28126111

[24] Berg M Veenhoven R. Income inequality and happiness in 119 nations: in search for an optimum that does not appear to exist. In: Greve B editor. Happiness and social policy in Europe. London: Edward Elgar Publishing (2010). doi: 10.4337/9781781000731.00017

[25] Jivraj S Nazroo J Vanhoutte B Chandola T. Aging and subjective well-being in later life. J Gerontol Series B-Psycholog Sci Soc Sci. (2014) 69:930–41. doi: 10.1093/geronb/gbu006, PMID: 24569002 PMC4296137

[26] Allard-Poesi F Matos LBS Massu J. Not all types of nature have an equal effect on urban residents’ well-being: a structural equation model approach. Health Place. (2022) 74:102759. doi: 10.1016/j.healthplace.2022.102759, PMID: 35255415

[27] Berenbaum MR. Proceedings of the National Academy of Sciences —its evolution and adaptation. Proc Natl Acad Sci USA. (2019) 116:704–6. doi: 10.1073/pnas.1821201116, PMID: 30617083 PMC6338832

[28] D’Ambrosio C Jäntti M Lepinteur A. Money and happiness: income, wealth and subjective well-being. Soc Indic Res. (2020) 148:47–66. doi: 10.1007/s11205-019-02186-w

[29] Kakwani NC. Measurement of tax progressivity: an international comparison. Econ J. (1977) 87:71. doi: 10.2307/2231833

[30] Yitzhaki S. On an extension of the Gini inequality index. Int Econ Rev. (1983) 24:617–28. doi: 10.2307/2648789

[31] Willis GB García-Sánchez E Sánchez-Rodríguez Á García-Castro JD Rodríguez-Bailón R. The psychosocial effects of economic inequality depend on its perception. Nat Rev Psychol. (2022) 1:301–9. doi: 10.1038/s44159-022-00044-0

[32] Roodman D. Fitting fully observed recursive mixed-process models with cmp. Stata J. (2011) 11:159–206. doi: 10.1177/1536867X1101100202

[33] Karlson KB Holm A Breen R. Comparing regression coefficients between same-sample nested models using logit and Probit: a new method. Sociol Methodol. (2012) 42:286–313. doi: 10.1177/0081175012444861

[34] Sun J. Income inequality, sense of fairness in distribution, and happiness. Econ. (2016) 1:42–9. doi: 10.16158/j.cnki.51-1312/f.2016.01.005

[35] Xu W Sun H Zhu B Bai W Yu X Duan R. Analysis of factors affecting the high subjective well-being of Chinese residents based on the 2014 China family panel study. Int J Environ Res Public Health. (2019) 16:2566. doi: 10.3390/ijerph16142566, PMID: 31323796 PMC6678496

